# Disease Progression-Dependent Expression of CD200R1 and CX3CR1 in Mouse Models of Parkinson’s Disease

**DOI:** 10.14336/AD.2019.0615

**Published:** 2020-03-09

**Authors:** Le Wang, Yang Liu, Shuxin Yan, Tianshu Du, Xia Fu, Xiaoli Gong, Xinyu Zhou, Ting Zhang, Xiaomin Wang

**Affiliations:** ^1^Department of Neurobiology, Center of Parkinson Disease Beijing Institute for Brain Disorders, Beijing Key Laboratory on Parkinson Disease, Key Laboratory for Neurodegenerative Disease of the Ministry of Education, Beijing Key Laboratory of Neural Regeneration and Repair, Capital Medical University, Beijing, China.; ^2^Department of Physiology and Pathophysiology, Capital Medical University, Beijing, China.

**Keywords:** microglia, Parkinson’s disease, CD200, CD200R1, CX3CL1, CX3CR1

## Abstract

Microglial activation is an important contributor to the pathogenesis of Parkinson’s disease (PD). Microglia are tightly and efficiently regulated by immune checkpoints, including CD200-CD200R1 and CX3CL1-CX3CR1. Understanding the involvement of these checkpoints in disease progression provides important insights into how microglial activation contributes to PD pathology. However, so far, studies have produced seemingly conflicting results. In this study, we demonstrate that CD200R1 expression is down-regulated at both early and late stage of PD model, and CX3CR1 expression is down-regulated in early stage and recovered in late stage. In primary cultured microglia, CD200R1 and CX3CR1 expressions are both directly regulated by LPS or α-synuclein, and CD200R1 expression is more sensitively regulated than CX3CR1. In addition, CD200 knockout causes an increase in proinflammatory cytokine production and microglial activation in the midbrain. Remarkably, DA neurons in the substantial nigra are degenerated in CD200^-/-^ mice. Finally, activation of the CD200R with CD200Fc alleviates the neuroinflammation in microglia. Together, these results suggest that immune checkpoints play distinct functional roles in different stage of PD pathology, and the CD200-CD200R1 axis plays a significant role in nigrostriatal neuron viability and function.

Parkinson’s disease (PD) is a neurological disorder characterized by the classical motor features of parkinsonism associated with Lewy bodies and the loss of dopaminergic neurons in the substantia nigra (SN) [1]. Numerous studies have highlighted the potential role of microgliosis in PD [2]. Reactive microglia are observed in postmortem brain samples of PD patients [3, 4]. The ongoing activation of microglia in PD has been confirmed using positron emission tomography (PET) imaging [5, 6].

Microglia are important modulators of the immune response in the CNS. In the presence of immune stimuli, microglia initiate a defense program, which should be tightly regulated to avoid excessive damage to surrounding tissues. The regulatory mechanisms include direct contact with neighboring neurons by immune checkpoint signals [7]. CX3CL1-CX3CR1 and CD200-CD200R1 pathways are the main inhibitory pathways that lead to the inactivation of microglia and keep them in a resting state [8]. CX3CR1 deficient mice showed exacerbated neuroinflammation and neurodegeneration in PD models [9, 10]. Chen et al. found that a CD200R1-blocking antibody injected into the striatum exacerbates microglial activation and dopaminergic neuro-degeneration in a rat model of 6-OHDA-induced PD [11]. These findings provide insights into the essential roles of these immune checkpoints in microglial activation of PD.

CX3CL1 is an essential chemokine, for regulating adhesion and chemotaxis through binding to its receptor (CX3CR1), which plays a crucial role in the crosstalk between microglia and neurons by direct or indirect ways in the CNS [12]. CX3CL1-CX3CR1 axis regulates microglial activation, neuronal survival and synaptic function by controlling the release of inflammatory cytokines and synaptic plasticity in the course of the neurological disease. The multiple functions of CX3CL1-CX3CR1 make it exert neuroprotective or neurotoxic effects, which determines the PD or neuroinflammation pathogenesis [12, 13]. However, the role of CX3CL1-CX3CR1 remains controversial during pathogenesis and progression of PD or neuroinflammation. Morganti reported that acute exposure to MPTP fails to affect the protein level of CX3CR1 [14]. Sun reported that the injection of MPP^+^ into the unilateral substantia nigra induces an increase in CX3CR1 expression [15]. Wynne showed that intraperitoneal lipopolysaccharide (LPS) injection causes a marked decrease in CX3CR1 expression [16].

CD200 and its receptor (CD200R1), expressed mainly in the membrane of neurons and microglia, respectively, also have been thought to involve in communications between neurons and microglia in the CNS expect CX3CL1-CX3CR1. CD200 interacts in a direct cell-to-cell manner with CD200R1, can inhibit microglial activation and keep “resting” to maintain brain homeostasis. Due to the important role of microglial activation in the pathogenesis and progression of PD, the CD200-CD200R dysfunction has been shown to be possibly involved in PD through microglial activation [17-19]. In addition, using CD200-CD200R signaling pathways as target sites to regulate microglia in PD, not only the toxic effect of microglial activation on neurons should be concerned, but also the regulation effect of neurons on microglia [19]. CD200R1 is consistently downregulated in MPTP models and LPS models [20-22]. However, CD200 has been reported to be upregulated and downregulated in LPS, spinal cord injury and PD models [20, 23]. These conflicting findings have raised concerns about the model-specific or disease progression-dependent expression and function of these checkpoints.

In this study, we assessed the expression of CX3CR1 and CD200R1 in the early and late stages of chronic progression in intraperitoneal LPS-injected mice and rAAV-hSYN unilaterally injected mice, which are both chronic model of PD, as well as in primary cultured microglia. We also investigated the functional role of the CD200-CD200R1 axis in neuroinflammation and neurodegeneration in CD200^-/-^ mice.

## METHODS AND MATERIALS

### Animals

All animal experimental procedures were approved by the Institutional Animal Care and Use Committee of the Capital Medical University, Beijing, China and were performed in accordance with the “Regulations for the Administration of Affairs Concerning Experimental Animals (the State Science and Technology Commission, China, 1988).” Male C57BL/6J mice (18 to 22 g) were purchased at the age of 7-8 weeks from Beijing Vital River Laboratory Animal Technology Co., Ltd. Transgenic CD200 knockout (CD200^-/-^) mice were purchased from IDMO and were maintained on a C57BL/6J background. Briefly, the deletion of the CD200 gene was achieved by a CRISPR-Cas9-mediated gene cleavage system. Cas9 mRNA and gRNA were synthesized using in vitro transcription and were used for microinjection. Sequencing analysis revealed that the CD200^-/-^ mice had a 1-bp deletion and a 2-bp deletion. The mice were housed under a 12:12-h light/dark cycle at 20°C to 23°C with free access to food and water.

#### 2.2 Primary cultured microglia

Primary rat microglial cells were prepared as described previously. Briefly, primary cultured microglia were prepared from the cerebral cortices of Sprague-Dawley rats (postnatal days 0-1) by stripping the meninges and blood vessels by mild mechanical trituration. The isolated cells were seeded in 175-cm^2^ culture flasks (3.5 brains per flask) in DMEM/F-12 containing 10% fetal bovine serum, 1% penicillin, and streptomycin. The cultures were maintained at 37°C in a humidified atmosphere of 5% CO_2_/95% air. Fourteen days later, the microglia were isolated as described previously from astrocytes by shaking the flasks at 180 rpm for 2 h. The purity of the enriched microglia was >95%, as identified by Iba-1 (1:800, Wako) immunocytochemical staining[24, 25].

The primary microglial cells were pretreated for 30 min with CD200Fc (2.5 μg/ml) and then administered different doses of LPS, human monomeric alpha-synuclein (α-syn)-purified protein or phosphate-buffered saline (PBS; Origene) for 24 hours.

#### 2.3 Surgical operations and drug treatment

All surgical procedures were performed under general anesthesia after an intraperitoneal (i.p.) injection of pentobarbital (50 mg/kg). The mice were injected with a single dose of LPS (5 mg/kg, i.p.) or 0.9% normal saline (N.S.) and sacrificed for subsequent analysis. We chose 9 hours after LPS injection as an early stage of LPS induced neuroinflammation and 10 months post injection as the late stage.

rAAV9-CMV-hSYN-3FLAG-WPRE and rAAV9-CMV-3FLAG-WPRE (3.67×10^13^ viral genomes/ml) were purchased from ViGene Biosciences. Wild-type mice were deeply anesthetized with pentobarbital and unilaterally injected with 2 μl of rAAV9-hSYN or rAAV9-3FLAG into the right substantia nigra pars compact (SNpc) to establish an α-syn-overexpressing PD model. The coordinates were as follows: -3.2 mm from bregma, 1.2 mm from the midline, and -4.6 mm from the dura.

### Protein extraction and Western blot analysis

Following treatment, the mice were anesthetized with pentobarbital. The midbrain tissue was isolated separately after 0.9% saline perfusion. Protein was extracted from cells or tissue with RIPA buffer (Beyotime) containing protease and phosphatase inhibitor cocktails (Roche) on ice. After sonication at 40% max power in 8-s bursts, the homogenates were centrifuged at 12,000×g for 20 min at 4°C, and the supernatant fraction was collected for analysis. Protein concentrations were determined using a BCA kit (Thermo Fisher Scientific). The proteins were resolved on 10% SDS polyacrylamide gels and transferred to PVDF membranes (Millipore). After blocking with 5% nonfat milk, the membranes were incubated overnight at 4°C with the following primary antibodies: mouse anti-GAPDH (1:10,000; Sigma), goat anti-CD200R1 (1:500; Santa Cruz), and rabbit anti-CX3CR1 (1:500; Abcam). The membranes were then washed with PBST (0.5% Tween-20 in PBS) and incubated with an IRDye 700- or 800-labeled secondary antibody (1:10,000, Rockland Immunochemicals). Detection was performed using the Odyssey infrared imaging system (LI-COR Biotechnology).

### RNA isolation and real-time PCR (RT-PCR)

Total RNA was isolated from primary microglia or tissue lysed in 1 mL of TRIzol reagent (Invitrogen, AM9738) in a 35-mm diameter dish, according to the recommended protocol. Cell or tissue lysates were transferred to tubes, and 0.2 mL of chloroform was added for total RNA extraction. The samples were centrifuged at 12,000×g for 15 min at 4°C. The RNA was in the aqueous phase, and the aqueous phase was transferred to a clean tube where the RNA was precipitated by the addition of isopropyl alcohol and centrifugation at 12,000×g for 10 min at 4°C. The supernatant was removed, and the RNA pellet was washed once with 75% ethanol, dried for 10 min, and dissolved in RNase-free water.

The total RNA was reverse transcribed into complementary DNA (cDNA) using the FastQuant RT kit (TIANGEN). Quantitative real-time RT-PCR was performed in triplicate using a Stratagene Mx3000P system (Agilent). A SYBR green DNA qPCR kit (Agilent) was used for real-time PCR analysis. The primer sequences that were employed are listed in Table 1. The GAPDH gene was used as an internal control to normalize the expression of the target gene. Target gene expression was calculated relative to the internal control, and analysis was performed with the formula 2^-ΔΔCT^. The mean CT of triplicate measures was calculated for each sample.

### Enzyme-linked immunosorbent assay (ELISA)

Microglial culture medium was collected at 24 h after LPS or α-syn stimulation. IL-1β and IL-6 concentrations in primary cultured microglia medium were quantified using traditional standard ELISA technique. ELISA analyses were performed by using ELISA kits in accordance with the manufacturer’s suggested protocol (ExCell Bio).

### Immunofluorescent staining and confocal microscopy

Primary rat microglial cells were seeded on poly-l-lysine-coated coverslips in 24-well plates and treated with CD200Fc, LPS and α-syn. Twenty-four hours later, the cells were fixed with 4% paraformaldehyde for 30 min, washed in PBS, and permeabilized with 0.3% Triton X-100 in PBS for 10 min at room temperature. After blocking with 10% normal horse serum for 1 h, the cells were incubated with goat anti-CD200R1 (1:100; Santa Cruz) overnight at 4°C. The next day, they were incubated with an Alexa Fluor 594-conjugated (1:500; Life Technologies) and/or an Alexa Fluor 488-conjugated secondary antibody (1:500; Life Technologies) for one hour at room temperature.

Brain tissue sections were prepared as previously described [26]. Briefly, nigral coronal sections (40 μm) from frozen paraformaldehyde-fixed brains were collected serially and stored at -20°C in tissue collection solution (50% 0.01 M PBS/50% glycerol). The sections were permeabilized in 0.01 M PBS containing 0.3% Triton X-100, blocked in 5% normal horse serum, and incubated overnight at 4°C with mouse anti-tyrosine hydroxylase (TH) (1:?2000, Sigma), rabbit anti-Iba-1 (1:500, Wako), rabbit anti-TH (1:1000, Novus) and mouse anti-human α-syn (1:500, Santa Cruz), followed by incubation with a secondary antibody as described above and imaged with a confocal microscope (Leica TCS SP8).

### Immunochemistry staining and the stereological quantification of nigral TH-positive cells

Mouse perfusion, tissue processing, and primary antibody incubation were performed as described above. The next day, the sections were incubated with a biotinylated secondary antibody (Vector Laboratories) for 60?min. The antibody was detected with an avidin/biotin complex (Vector Laboratories) and visualized with 3,3-diaminobenzidine (Sigma). The sections were imaged with a bright-field microscope (Olympic). To count the TH-immunopositive cells in the SNpc, a total of five TH-labeled sections containing the SNpc were selected for each animal (5-12 mice per group). Using a bright-field Olympic microscope, the boundaries of the SNpc area were traced. The number of TH-immunoreactive cells within the traced area was counted by stereology using Stereo Investigator software (MBF Bioscience), as described previously [27].

**Table 1:** Primers used for RT-qPCR.

Gene	Gen-Bank ID	Sequences (5’ - 3’)
mouse-CD200	NM_010818.3	Forward: CTGTGAGGGATTTGACTTTTTGC Reverse: CCGAGGCACTCGACTTCCT
mouse-CD200R1	NM_021325.3	Forward: GGAAAACCAGAAAACCGAAATG Reverse: CCCCCATATTAAGAGCACTGCTA
mouse-TNF-α	NM_013693.3	Forward: CCAGTGTGGGAAGCTGTCTT Reverse: AAGCAAAAGAGGAGGCAACA
mouse-IL-1β	NM_008361.4	Forward: CTGGTGTGTGACGTTCCCATTA Reverse: CCGACAGCACGAGGCTTT
mouse-CX3CL1	NM_009142.3	Forward: TGGCTTTGCTCATCCGCTAT Reverse: CTGTGTCGTCTCCAGGACAAT
mouse-CX3CR1	NM_009987.4	Forward: CAAGCTCACGACTGCCTTCT Reverse: CTGCACTGTCCGGTTGTTCA
mouse-GAPDH	NM_001289726.1	Forward: AGAACATCATCCCTGCATCC Reverse: CACATTGGGGGTAGGAACAC
rat-CD200R1	NM_023953.1	Forward: GTCCTTGGATGGGCATTTA Reverse: TGCGGAGATTCACCACAA
rat-CX3CR1	NM_133534.1	Forward: TCCCTTGTCTTCACGTTCGG Reverse: ACAAAGAGCAGGTCGCTCAA
rat-IL-1β	NM_031512.2	Forward: AAATGCCTCGTGCTGTCTGA Reverse: TGGAGAATACCACTTGTTGGC
rat-IL-6	NM_012589.2	Forward: GCCCACCAGGAACGAAAGTC Reverse: TGGCTGGAAGTCTCTTGCGG
rat-PPARγ	NM_013124.3	Forward: GGAGATCCTCCTGTTGACCC Reverse: TGGTAATTTCTTGTGAAGTGCTCA
rat-C/EBPβ	NM_024125.5	Forward: ACCACGACTTCCTTTCCGAC Reverse: TAACCGTAGTCGGACGGCTT
rat-GAPDH	NM_017008.4	Forward: AGAACATCATCCCTGCATCC Reverse: CACATTGGGGGTAGGAACAC

### Rotarod test and cylinder test

Rotarod tests were performed to evaluate motor coordination and learning. The mice were trained with accelerating speeds for 3 consecutive days immediately before the first test and were subjected to a total of 3 trials on the rotarod with accelerating speeds (4-50 rpm), a maximal duration of 5 min, and an interval of 1 h between trials. The mean latency to fall off the rotarod was recorded.

Cylinder tests were performed to assess forelimb lateralization. The mice were placed individually in a transparent/plexiglass cylinder (10 cm in diameter, 17.5 cm high) surrounded by two mirrors so that the animal could be observed from all directions. The total number of ipsilateral paws, contralateral paw and bilateral paw touches on the glass was recorded for 5 min. The data are shown as the percentage of contralateral contacts and were calculated by the following equation: contralateral touches/ (contralateral touches + ipsilateral touches + bilateral touches).

Each behavioral test was performed by the different individual in the same environment and similar time. To test the influence of α-syn overexpression on motor behavior at different time, a one-way no-repeated measures ANOVA was performed to confirm if significant difference from the control group was observed over time.

### Microglial Isolation

Mice were sacrificed and perfused with N.S., and brains (except the cerebellum) were separated and digested by dispase II, DNaes I and papain, strained and dissociated into single cells through a 40-μm filter, and centrifuged on a 30% isotonic Percoll (GE Healthcare) solution for 10 min [28]. After the myelin and debris were removed, most of the remaining sample (except the pellet) was used for immunomagnetic separation by anti-CD11b magnetic particles - DM (BD Biosciences).

### Statistical Analysis

All data are expressed as the means ± SEM. The data were analyzed with GraphPad Prism 7.0 software (GraphPad Software). Student's unpaired t test was used to compare two data sets. For multigroup comparisons, one-way ANOVA followed by Newman-Keuls test was used. For all analyses, statistical significance was accepted when *P* < 0.05.

**Figure 1.**
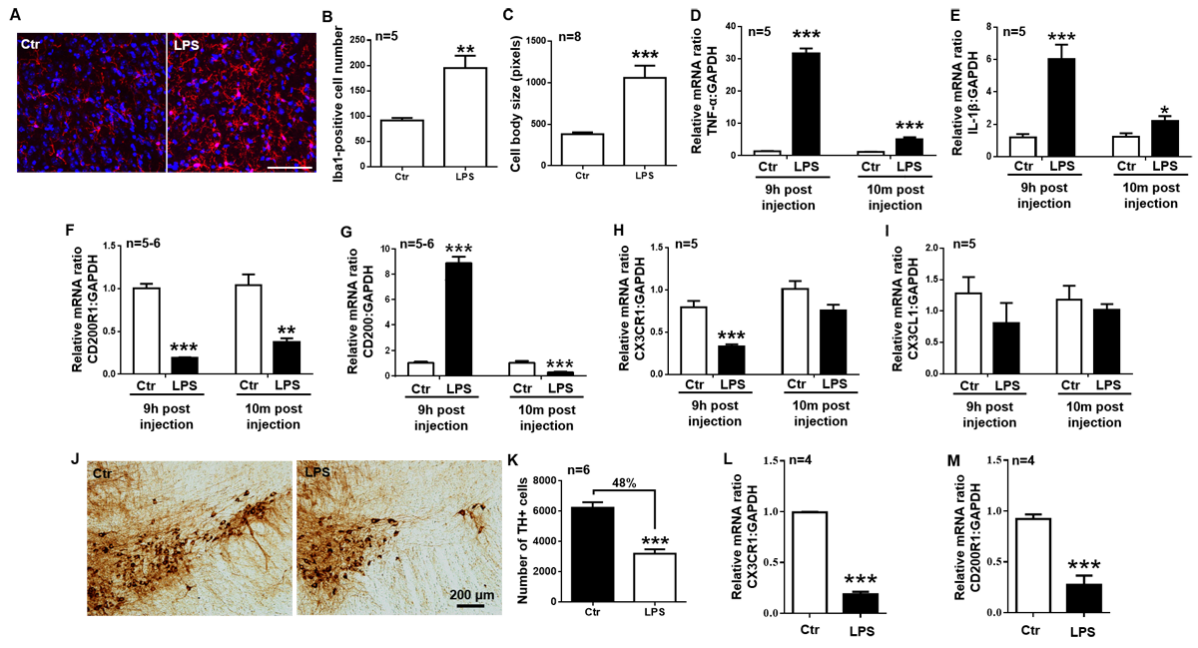
The temporal expression of CD200R1 and CX3CR1 in the early and late stages of LPS-induced PD. A single dose of LPS (5 mg/kg, i.p.) or N.S. control (ctr) was administered. (A) Representative immunofluorescent images of Iba1^+^ microglia 3 h after LPS injection (5 mg/kg, i.p.). Scale bar = 75 μm. Red, Iba1; blue, Hoechst. (B, C) The quantification of the number (B) and cell body size (C) of Iba1^+^ cells 3 h after LPS injection (n = 5-8 per group, 10 cells per mouse). The mice were sacrificed at different time points after LPS or control injection (9 hours or 10 months) for the analysis of TNF-α (D), IL-1β (E), CD200R1 (F), CD200 (G), CX3CR1 (H) and CX3CL1 (I) levels in the midbrain by RT-qPCR (n = 5-6 per group). (J and K) Representative immunochemical images (J) and the quantification of the number of TH^+^ dopamine neurons (K) in the SNpc 10 months after LPS injection. Scale bar = 200 μm (n = 6 per group). Microglia were isolated from the brains (excluding the cerebellum) 9 hours after LPS or control injection for the analysis of CX3CR1 (L) and CD200R1 (M) levels by RT-qPCR (n = 4 per group). The data are expressed as the mean ± SEM. * *p* < 0.05, ** *p* < 0.01 and *** *p* < 0.001 versus the ctr group, Student’s *t* test.

## RESULTS

### The temporal expression of CD200R1 and CX3CR1 are different in early and late stages of systemic LPS injection model

To observe CD200R1 and CX3CR1 expression in chronic inflammation, we administered a single dose of LPS (5 mg/kg, i.p.), which is thought to activate microglia and produce proinflammatory cytokines, resulting in a delayed and progressive loss of DA neurons in the SN [29, 30], to wild-type C57BL/6 mice. The mice were then sacrificed at 9 h (the early stage) and 10 m (the late stage) post injection to analyze changes in microglial activation, cytokine production, and CD200R1 and CX3CR1 expression in the midbrain. The number of microglia was increased, and the cell bodies were enlarged 3 h after LPS injection, indicating that microglia were activated after LPS challenge (Fig. 1A-C). TNF-α and IL-1β mRNA increased significantly 24.9-fold and 5.06-fold, respectively, 9 h post injection, and these increased levels nearly recovered (to 4.73-fold and 1.75-fold greater levels) 10 m post injection (Fig. 1D, E). Meanwhile, both CD200R1 and CX3CR1 expression decreased significantly 9 h after LPS injection, but only CD200R1 expression was still decreased 10 m after LPS injection; CX3CR1, however, which was alleviated to a similar level as that in the control group (Fig. 1F, H). To evaluate ligand expression, we found that CD200 was increased significantly 9 h after injection but was decreased 10 m after injection (Fig. 1G). No obvious changes in CX3CL1 expression were observed (Fig. 1I). We also counted the number of TH^+^ dopaminergic neurons by stereology and found that the number was decreased by 48% 10 m after LPS injection, indicating a chronic effect of LPS on dopaminergic neurons in the SN (Fig. 1J, K). These results indicate that CD200-CD200R1 is involved in both the early and late stages of LPS-induced neuroinflammation and that CX3CL1-CX3CR1 is only involved in the early stage.

To determine CD200R1 and CX3CR1 expression in microglia, we isolated microglia from the brains of mice injected with or without LPS by immunomagnetic separation. To validate the efficiency of the separation, we tested the signature gene expression of microglia, neurons, astrocytes and oligodendrocytes in CD11b-positive and CD11b-negative extracts. We found that CX3CR1 was exclusively expressed in CD11b^+^ extracts, while GFAP, NeuN, Olig2 was expressed in CD11b^-^ extracts, indicating the successful purification of microglial cells from the brain (Supplemental Fig. 1). Nine hours after LPS injection, CD200R1 and CX3CR1 expression was decreased significantly to a similar level as that observed in the mouse midbrain tissue (Fig. 1L, M).

**Figure 2.**
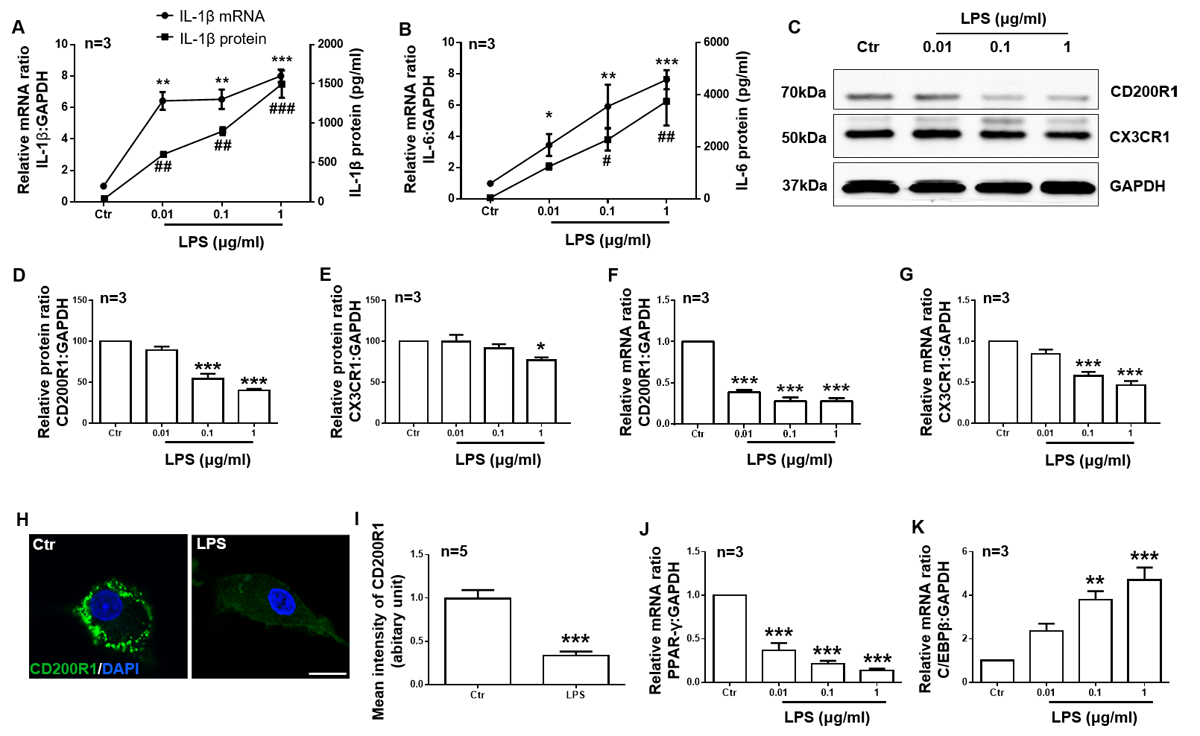
CD200R1 and CX3CR1 expression is downregulated by LPS in microglia. Primary cultured microglia were stimulated with different concentrations of LPS (0.01, 0.1, and 1 μg/ml). Twenty-four hours later, IL-1β (A) and IL-6 (B) expression was tested by RT-qPCR and ELISA. CD200R1 (C, D) and CX3CR1 (C, E) protein expression was tested by RT-qPCR and Western blot. CD200R1 (F) and CX3CR1 (G) mRNA expressions were observed by RT-qPCR. (H) Representative immunofluorescent images of CD200R1 expression in microglia 24 h after LPS (1 μg/ml) treatment. Scale bar = 10 μm. Green, CD200R1; blue, Hoechst. (I) The quantification of CD200R1 staining intensity in microglia (n = 5 per group). PPAR-γ (J) and C/EBPβ (K) expression was tested by RT-qPCR. The data are expressed as the mean ± SEM (n = 3 per group). * *p* < 0.05, ** *p* < 0.01 and *** *p* < 0.001 versus the ctr group, Student’s t test or one-way ANOVA. ## *p* < 0.05, ## *p* < 0.01 and ### *p* < 0.001 versus the ctr group, one-way ANOVA.

**Figure 3.**
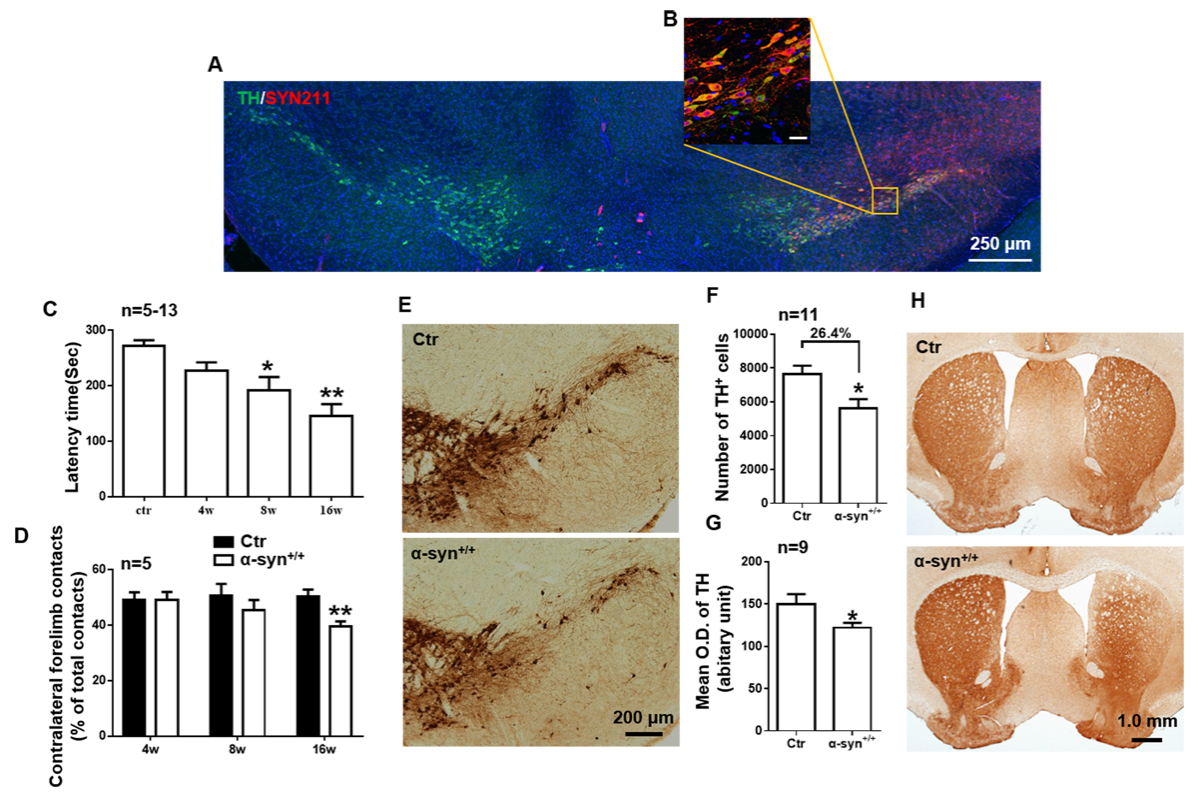
The characterization of the rAAV-hSYN-injected mouse model of PD. Two-month-old C57BL/6 mice received a unilateral stereotactic injection of rAAV9-hSYN into the right SNpc to generate a mouse model of PD. Two weeks postinjection, (A) the mice were sacrificed, and brain slices were stained with a human-specific α-syn antibody (αsyn 211, red). Green, TH. Scale bar = 250 μm. (B) A higher magnification image showing TH^+^ neurons expressing exogenous α-syn. Scale bar = 25 μm. The rotarod test (C) and cylinder test (D) were performed 4, 8 and 16 weeks after rAAV-hSYN injection (n = 5-13 per group). Representative immunochemical images (E) and the quantification of the number of TH^+^ neurons (F) in the SNpc at 8 weeks after rAAV-hSYN injection (n = 11 per group). Scale bar = 200 μm. (G) Quantification of changes in TH immunoreactivity in the ipsilateral striatum of CD200 ^-/-^ and WT mice in a mouse PD model at 8 weeks after intra-SNpc infection (n = 11 per group). (H) Representative immunochemical images of TH immunostaining of the striatum. Scale bar = 1.0 mm. The data are expressed as the mean ± SEM. * *p* < 0.05 and *** *p* < 0.001 versus the ctr group, Student’s t test or one-way ANOVA.

Next, we used primary cultured microglia stimulated with different concentrations of LPS (0.01, 0.1, 1 μg/ml) to further confirm that CD200R1 and CX3CR1 expression in microglia is directly regulated by LPS. Twenty-four hours after LPS stimulation, we found that both IL-1β and IL-6 production was increased significantly in a dose-dependent manner (Fig. 2A, B). Both CD200R1 and CX3CR1 expression decreased, but, compared to CX3CR1 expression, CD200R1 expression decreased more significantly (Fig. 2C). The protein level of CD200R1 decreased to 54.04% of the baseline level by 0.1 μg/ml LPS administration and to 40.35% of the control level by 1 μg/ml LPS, while CX3CR1 decreased to 77.03% of the control level by 1 μg/ml LPS (Fig. 2D, E). Similar results were observed for the mRNA levels of CD200R1 and CX3CR1 expression (Fig. 2F, G). We also performed immunofluorescence staining of CD200R1 in microglia after 1 μg/ml LPS treatment, and the data further validated the decrease observed in CD200R1 expression after LPS stimulation (Fig. 2H, I). Since it has been reported that peroxisome proliferator-activated receptor gamma (PPARγ) and CCAAT enhancer-binding protein beta (C/EBPβ) bind the CD200R1 gene promoters to modulate CD200R1 expression [31, 32], we tested the expression of these factors and found that PPARγ mRNA expression was significantly decreased while C/EBPβ mRNA expression was significantly increased by LPS treatment (Fig. 2J, K). These data confirm that the expression of both CD200R1 and CX3CR1 is downregulated by LPS directly in microglia and that CD200R1 expression decreases more significantly.

**Figure 4.**
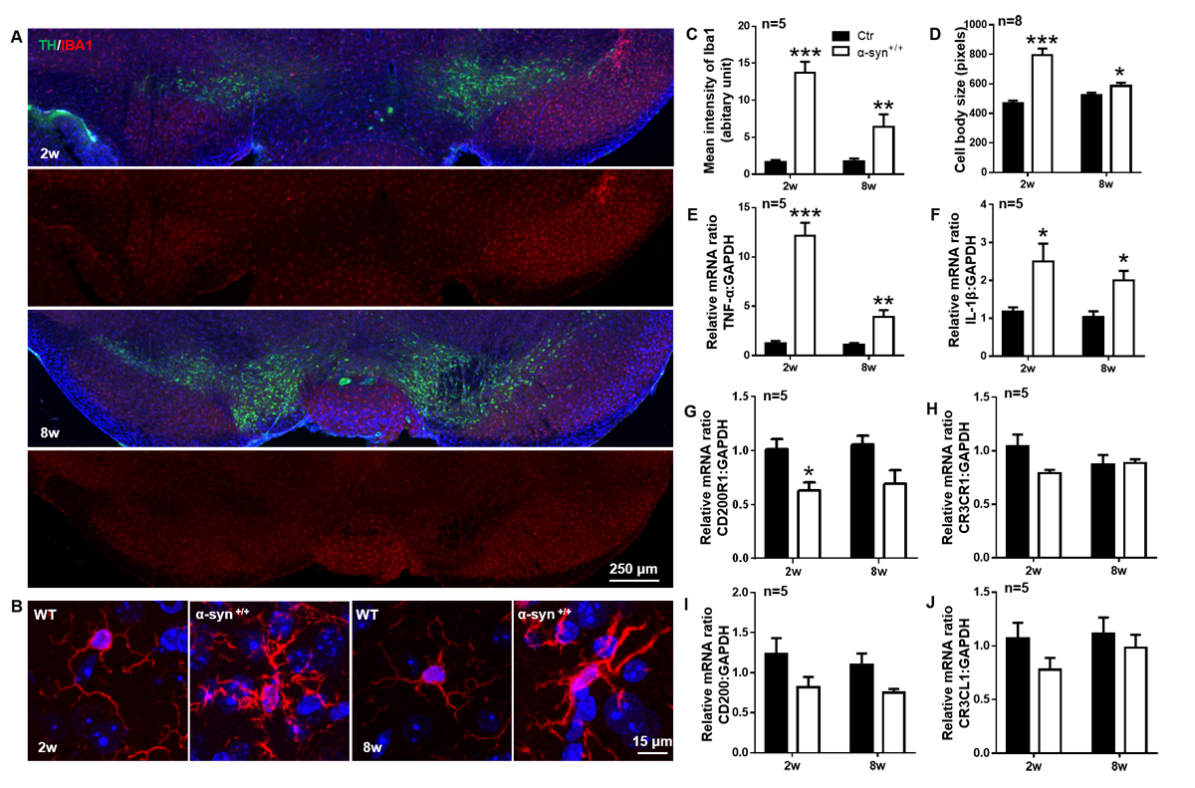
The temporal expression of CD200R1 and CX3CR1 in the early and late stages of PD. C57BL/6 mice received a unilateral stereotactic injection of recombinant AAV encoding human full-length α-syn (rAAV9-hSYN) (3.67×10^13^ viral genomes/ml) into the right SNpc. The mice were sacrificed 2 weeks and 8 weeks postinjection. (A) Representative immunofluorescent images of Iba1^+^ microglia in the SNpc of a mouse model of PD. Scale bar = 250 μm. Red, Iba1; green, TH; blue, Hoechst. (B) Higher magnification images of Iba1+ microglia in the ipsilateral SN of the control group and PD group. Scale bar = 15 μm. Red, Iba1; blue, Hoechst. (C, D) The quantification of the Iba1 staining intensity (C) and cell body size (D) of Iba1^+^ cells (n = 5-8 per group, 10 cells per mouse). The mice were sacrificed at different time points after virus injection (2 weeks or 8 weeks) for the analysis of TNF-α (E), IL-1β (F), CD200R1 (G), CX3CR1 (H), CD200 (I) and CX3CL1 (J) levels in the midbrain by RT-qPCR. The data are expressed as the mean ± SEM (n = 5 per group). * *p* < 0.05, ** *p* < 0.01 and *** *p* < 0.001 versus the ctr group, Student’s t test.

### The temporal expression of CD200R1 and CX3CR1 are different in the early and late stages of in a rAAV-hSYN model of PD

Because elevated inflammatory signaling and microglial activation in the midbrain may contribute to neuronal susceptibility to Parkinsonian degeneration, it is intriguing to investigate whether CD200R1 and CX3CR1 expression is decreased in a model of PD. C57BL/6 mice received a unilateral stereotactic injection of recombinant AAV encoding human full-length alpha-synuclein (rAAV9-hSYN) (3.67×10^13^ viral genomes/ml) into the right SNpc to generate a mouse model of PD. Two weeks after injection, α-syn was widely expressed in the dopaminergic neurons of the SNpc, as indicated by double-staining for TH and α-syn (Fig. 3A, B). The motor behavior of the mice injected with rAAV-hSYN was impaired significantly 8 wks post injection, as tested by rotarod, and 16 wks post injection, as determined by both rotarod (Fig. 3C) and cylinder test (Fig. 3D). The number of dopaminergic neurons was decreased by 26.4% 8 wks post injection (Fig. 3E, F). Furthermore, we quantified TH immunopositive nerve terminals by densitometry and found it is also decreased in the striatum (Fig. 3G, H).

Microglial activation was evaluated by Iba-1 staining in the PD mice. We found that the intensity of Iba-1 expression was increased significantly by 8.31-fold at the early stage (2 wks post injection) and decreased to 3.64-fold at late stage (8 wks post injection) (Fig. 4A-C). T The cell body size was increased by 1.71-fold 2 wks post injection and by 1.13-fold 8 wks post injection (Fig. 4A, B, D). TNF-α and IL-1β mRNA expression was increased significantly by 9.90-fold and 2.13-fold, respectively, 2 wks post injection and was recovered to levels that were 3.48-fold and 1.93-fold greater than those of the control, respectively, 8 wks post injection (Fig. 4E, F). These results indicate a significant activation of microglia in the early stage of PD and insufficient activation in the late stage.

The expression of CD200R1 and CX3CR1, as well as that of their ligands, were decreased by 37.4% and 24.2%, respectively, 2 wks post injection, and this change was significant. However, 8 wks post injection, only CD200R1 and CD200 expression remained decreased by 34.65% and 31.56%, respectively, compared to that of the noninjected control animals. Despite the decrease of CD200R1 and CD200 expression was lack of significance, there is certainly a declining trend. (Fig. 4G, I). The expression of CX3CR1 recovered to nearly the same level as that of the noninjected control animals (Fig. 4H, J). These results indicate that CD200-CD200R1 is involved in both the early and late stages of rAAV-hSYN-induced PD and that CX3CL1-CX3CR1 is only involved in the early stage.

**Figure 5.**
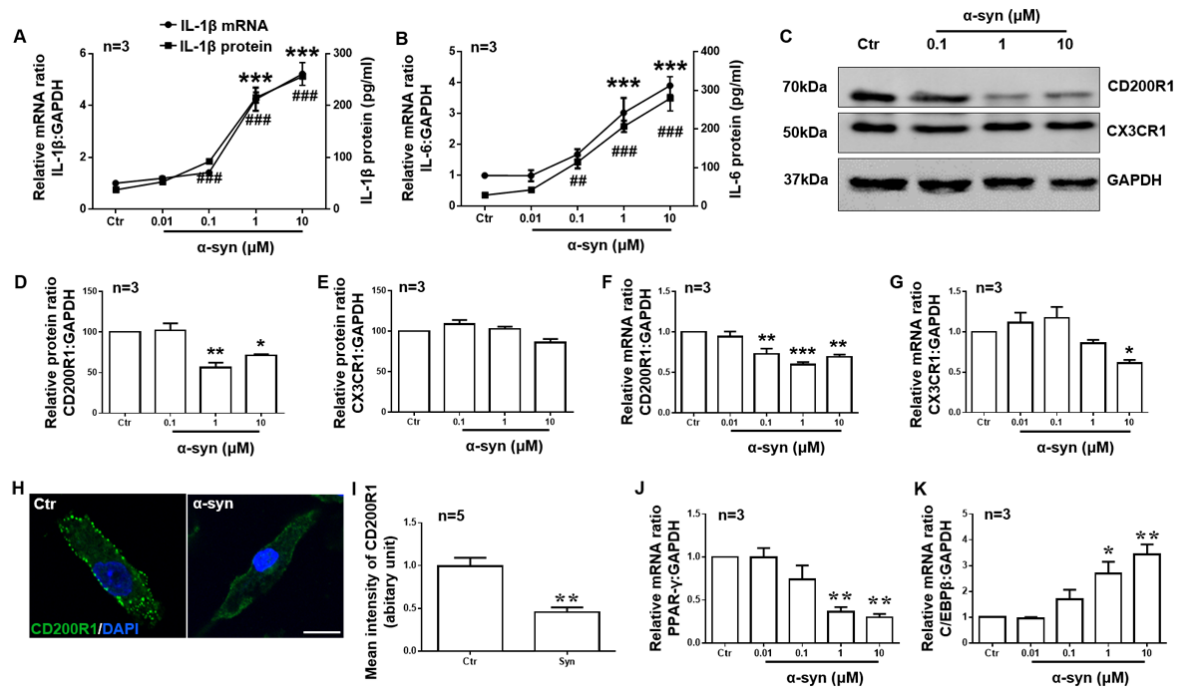
CD200R1 is more sensitively regulated by α-syn in microglia. Primary cultured microglia were prepared from the cerebral cortices of SD rats (P0) and stimulated with different concentrations of monomeric recombinant human α-syn protein (0.01, 0.1, 1 and 10 μM). After 24 h, IL-1β (A) and IL-6 (B) expression and release were evaluated by RT-qPCR and ELISA. CD200R1 (C, D) and CX3CR1 (C, E) expression was tested by RT-qPCR and Western blot. CD200R1 (F) and CX3CR1 (G) mRNA expressions were observed by RT-qPCR. (H) Representative immunofluorescent images of CD200R1 expression in microglia 24 h after α-syn (1 μM) stimulation. Scale bar = 10 μm. Green, CD200R1; blue, Hoechst. (I) The quantification of CD200R1 staining intensity in microglia (n = 5 per group). PPAR-γ (J) and C/EBPβ (K) expression was tested by RT-qPCR. The data are expressed as the mean ± SEM (n = 3 per group). * *p* < 0.05, ** *p* < 0.01 and *** *p* < 0.001 versus the ctr group, Student’s t test or one-way ANOVA. ## *p* < 0.01 and ### *p* < 0.001 versus the ctr group, one-way ANOVA.

We also investigated the direct effect of α-syn on CD200R1 and CX3CR1 expression in microglia. Twenty-four hours after α-syn treatment, the IL-1β and IL-6 levels were increased significantly in a dose-dependent manner (Fig. 5A, B). Importantly, the CD200R1 protein level was significantly decreased by 43.7% by 1 μM α-syn stimulation and by 28.5% by 10 μM α-syn stimulation (Fig. 5C, D), while CX3CR1 was not significantly decreased (decreased by 14.15%) by 10 μM α-syn stimulation (Fig. 5C, E). Similar results were observed for mRNA level of CD200R1 and CX3CR1 expression as evaluated by RT-qPCR (Fig. 5F, G). We further confirmed the decrease in CD200R1 expression after 1 μM α-syn stimulation by immunofluorescence (Fig. 5H, I). We also observed the expression of the upstream transcription factors PPARγ and C/EBPβ and found similar changes as those induced by LPS treatment (Fig. 5J, K). These results indicate that CD200R1 is more sensitively regulated by α-syn in microglia.

**Figure 6.**
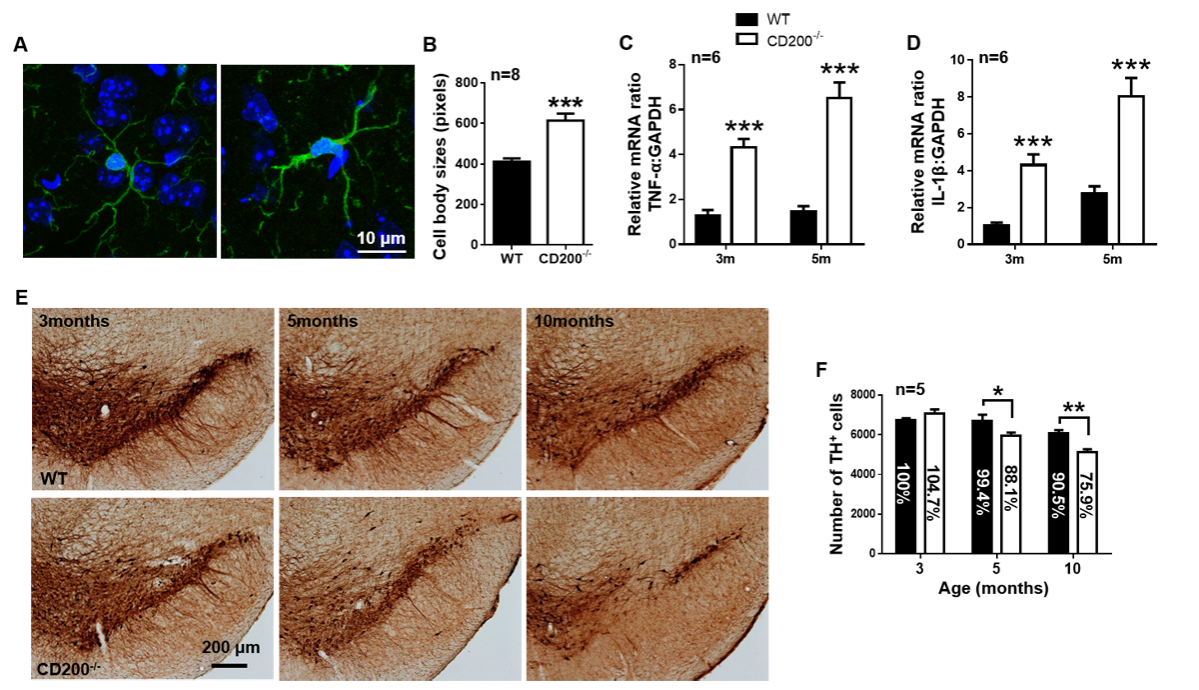
CD200 deficiency induces microglial activation and dopaminergic neuron death in SNpc. (A) Representative immunofluorescent images of Iba1^+^ microglia in the SN of CD200^-/-^ and WT mice at 3 months of age. Scale bar = 10 μm. Green, Iba1; blue, Hoechst. (B) The quantification of the cell body size of Iba1^+^ cells (n = 8 per group). TNF-α (C) and IL-1β (D) mRNA expression in the midbrain of 3- and 5-month-old CD200^-/-^ and WT mice, as detected by RT-qPCR (n = 6 per group). (E) Representative TH immunostaining images illustrating the morphology of DA neurons and the stereological quantification of TH^+^ neurons (F) in CD200^-/-^ and WT mice at 3, 5 and 10 months of age. Scale bar = 200 μm. (n = 5 per group). The data are expressed as the mean ± SEM. * *p* < 0.05, ** *p* < 0.01 and *** *p* < 0.001 versus the WT mice, Student’s t test.

### CD200 deficiency causes microglia activation and dopaminergic neuron death in the SNpc

Since CD200-CD200R1 is involved in both the early and late stages of neuroinflammation and PD, we next investigated whether CD200-CD200R1 deficiency has a chronic effect on dopaminergic neurons in the SNpc. We generated CD200^-/-^ mice using CRISPR-Cas9-mediated genome editing by introducing 1-bp and 2-bp deletions into the 3^rd^ exon of the murine CD200 gene (Supplementary Fig. 2A, B). We also tested CD200 expression in CD200^-/-^ mouse brain by RT-qPCR and western blot (Supplementary Fig. 2C, D).

Next, microglial activation was observed by morphological analysis. The cell body size increased by 1.49-fold in 3-month-old CD200^-/-^ mice (Fig. 6A, B). The expression of TNF-α and IL-1β mRNA was increased significantly by 4.44-fold and 2.91-fold, respectively, in 5-month-old CD200^-/-^ mice compared with their littermates (Fig. 6C, D). We further counted the number of dopaminergic neurons in the SNpc by stereology and found that it decreased significantly by 11.3% at 5 months of age and by 14.6% at 10 months of age (Fig. 6E, F). These results indicate that CD200 deficiency causes significant microglial activation and dopaminergic neuron loss in the SNpc.

### CD200Fc suppresses cytokine production and the decrease in CD200R1 expression induced by LPS or α-syn in microglia.

**Figure 7.**
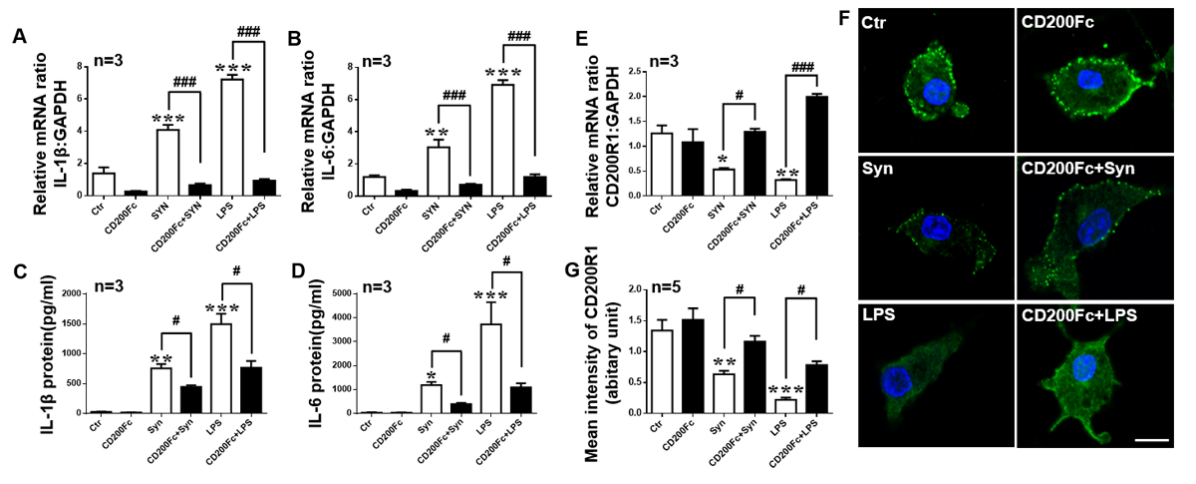
CD200Fc attenuates cytokine production and the decrease in CD200R1 induced by LPS or α-syn in microglia. Primary cultured microglia were pretreated with CD200Fc (2.5 μg/ml) for 30 min and then exposed to monomeric recombinant human α-syn protein (1 μM) or LPS (1 μg/ml) for 24 h. IL-1β and IL-6 expression was evaluated by RT-qPCR (A, B) and ELISA (C, D). (E) CD200R1 expression was tested by RT-qPCR (E) and immunofluorescent staining (F, G) in microglia treated with α-syn or LPS and treated with or without CD200Fc. Scale bar = 10 μm. Green, CD200R1; blue, Hoechst. (n = 5 per group). The data are expressed as the mean ± SEM (n = 3 per group). * *p* < 0.05, ** *p* < 0.01 and *** *p* < 0.001 versus the ctr group, one-way ANOVA. # *p* < 0.05 and ### *p* < 0.001 versus the α-syn or LPS group, one-way ANOVA.

To investigate whether CD200R1 activation can alleviate the cytokine production induced by LPS or α-syn in microglia, we added CD200Fc before LPS or α-syn treatment. We found that both IL-1β and IL-6 production was decreased significantly by CD200Fc pretreatment (Fig. 7A-D). Interestingly, CD200R1 expression was also rescued by CD200Fc administration (Fig. 7E-G). These results indicate that the CD200-CD200R1 axis is very efficient in downregulating cytokine release in microglia.

## DISCUSSION

In the present study, we observed CD200-CD200R1 and CX3CL1-CX3CR1 expression in the early and late stages of PD in mouse models of PD. In the LPS-injected mice and rAAV-hSYN-injected mice, CD200R1 expression was downregulated both in the early and late stages after injection. However, CX3CR1 expression was downregulated in the early stage and was recovered in the late stage after injection. In primary cultured microglia, we found that CD200R1 was directly downregulated by LPS or α-syn. However, CX3CR1 expression was downregulated by LPS and was unchanged by α-syn. Finally, we found that cytokine production and progressive dopaminergic neuron loss increased significantly in the CD200^-/-^ mice. In contrast, CD200Fc suppressed cytokine production and the decreased in CD200R1 induced by LPS or α-syn in microglia.

Neurodegenerative diseases are chronic pathological conditions that persist for many years. In rodents, the majority of studies aimed at investigating microgliosis in PD have been performed in acute models [33-36]. A more recent study investigated reactive microglia in rodents by viral vector-mediated a-synuclein overexpression targeted directly to nigrostriatal dopaminergic neurons [37-40]. A long time was required to provide overt dopaminergic degeneration. In the present study, the number of dopamine neurons was decreased by 26.4% at 8 wks post injection (Fig. 3F). Decressac and Marusela reported that the loss of dopaminergic neurons reaches a maximum of 67% and 82%, respectively, after 8 wks [26, 41]. It is also possible to produce rodent models of PD by the accumulation of proteinase K, which is insensitive a-synuclein aggregates [42]. In addition, we found the motor behavior of the mice injected with rAAV-hSYN was impaired significantly in both rotarod (Fig. 3C) and cylinder test (Fig. 3D), while the loss of striatal TH^+^ fibers was 19.1% (Fig. 3G). However, it is generally accepted that bradykinesia appear in PD when dopamine neurons loss exceeds a threshold, 70-80% of striatal nerve terminals and 50-60% nigral neurons death [43, 44]. Salvatore and Bezard found striatal TH or dopamine content decline are not critical factor in motor behavior impairment [44, 45]. Other reports suggested that TH^+^ neuron or dopamine loss in the SN contributes to be a central mechanism of motor behavior impairment, the critical threshold is 30%-50% dopamine neurons death [46-48]. In our study, TH^+^ neurons loss in the SNpc contributed to motor deficits at 8 wks because the virus is locally injected into SNpc. Besides, another cause of motor behavior impairment post injection is likely to be α-syn overexpression induced axonal and/or synaptic dysfunction in the surviving neurons [41, 49]. This model is promising for providing a deeper understanding of the pathogenic mechanisms of cellular dysfunction, including microgliosis, underlying PD.

The interaction of microglia with surrounding cells during disease progression is dynamic and heterogenous. In the early stage, microglia are significantly activated, as shown by the morphological changes and cytokine production in both the LPS- and rAAV-hSYN-injected models (Fig. 1 and Fig. 4). Two weeks after rAAV-hSYN injection, cytokine production reached a peak level, increasing 9.90-fold and 2.13-fold compared with that in the control group. This is consistent with the timeline reported by Theodore [40] and Su [50]. Inflammation is not the only response of microglial activation in the early stage of PD; it has also been shown that CD68 is increased 4 wks postinjection, indicating increased phagocytosis of microglia [40]. Inflammation impairs the ability of microglia to discriminate between dead and viable neurons for phagocytosis, leading to neuronal death in the early stage of the disease [51].

However, in the late state of PD, microglia are insufficiently activated. Morphological changes were attenuated, and cytokine production decreased (Fig. 4). Eight weeks after rAAV-hSYN injection, cytokine production increased by 3.48-fold and 1.93-fold compared with that in the rAAV-3FLAG control group. In this period, CD68 expression declined, and the accumulation of phosphorylated α-syn in the affected DA neurons increased [37, 40]. These results suggest that the microglial response is distinct during the late stage of the PD.

Microglial checkpoint mechanisms are also different in the early and late stages of PD. In the early stage, both CD200R1 and CX3CR1 expression decreased significantly. Nine hours post-LPS injection, CD200R1 was decreased by 81.02% compared with the level in the control group, and CX3CR1 decreased by 58.23%. Two weeks post rAAV-hSYN injection, CD200R1 decreased by 37.36% compared with the level in the control group, and CX3CR1 decreased by 24.18%. Interestingly, CD200 ligand expression was increased by 8.64-fold 9 h post LPS injection, but this was not observed for CX3CL1 expression. These results are consistent with a previous study showing that CD200 expression is increased significantly 4 h post-LPS injection [20] and another study showing that CX3CL1 expression is unaltered after LPS injection [16]. These results indicate that both CD200-CD200R1 and CX3CL1-CX3CR1 are involved in the checkpoint mechanisms regulating microglial phenotypes in the early stage of PD and that CD200-CD200R1 signaling is more sensitive. We then observed microglial activation in the CD200^-/-^ mice and found an increase in the production of proinflammatory cytokines, including TNF-α and IL-1β. More importantly, a progressive loss of DA neurons in the SNpc was observed in the CD200^-/-^ mice. Previous studies have reported that CD200-deficient mice show an accelerated microglial response that results in a more rapid onset of experimental autoimmune encephalomyelitis [52]. This is the first evidence that CD200 deficiency directly causes neuronal death in the SN.

In the late stage of PD, CD200 and CD200R1 remained at lower levels, similar to what was observed in the early stage. However, CX3CL1 and CX3CR1 expression in the rAAV-hSYN-injected mice recovered to nearly the same level as that in control mice (Fig. 4G, H). Ten months after LPS injection, when the number of TH-positive DA neurons was decreased by 48% of that in the control mice, CD200 expression decreased by 71.25% and CD200R1 expression decreased by 63.91%. However, CX3CL1 and CX3CR1 expression in the LPS-injected mice recovered to nearly the same level as that in the control mice (Fig. 1H, I). In a model of MPTP/P-induced chronic PD, Sung observed a significant decrease in CD200 and CD200R1 expression in the midbrain of the MPTP/P mice [21]. These results indicate that CD200-CD200R1 is involved in the late stage of PD pathology.

Recently, the single-cell RNA analysis of CNS immune cells in neurodegenerative conditions revealed disease-associated microglia (DAM). DAMs are characterized by the downregulation of microglial checkpoint genes, including CX3CR1, CD200R1 and TREM2 [53]. Accumulating evidence from human genetics studies has provided strong evidence that DAMs provide a protective innate immune response against AD pathology [54]. The remaining levels of CX3CL1 and CX3CR1 expression in the late stage of PD may limit the ability of microglia to protect the CNS. A recent study reported that TREM2 deficiency has a disease-progression-dependent effect on amyloid pathology by ameliorating amyloid pathology early but exacerbating it late in AD in APPPS1-21 mice [55]. These results indicate that blocking immune restraints can have both beneficial and detrimental consequences depending on the disease stage.

Microglia express dedicated pattern recognition receptors (PPRs) that sense microbial molecules, i.e., highly conserved pathogen-associated molecular patterns (PAMPs). Other PPRs in microglia detect damage-associated molecular patterns (DAMPs). DAMPs can be released by damaged cells, including misfolded proteins, aggregated peptides, and nucleic acids and are found under neurodegenerative conditions [56, 57]. In the present study, we observed that both the ligands, but the receptors of the inhibitory checkpoints were downregulated in a model of PD. Moreover, in cultured microglia, CD200R1 and CX3CR1 expression was directly downregulated by LPS. CD200R1 was more sensitive because it decreased significantly after 0.01 µg/ml LPS treatment and decreased 28.44% and 28.48% after 0.1 and 1 µg/ml LPS treatment, respectively. CX3CR1 expression decreased significantly by 42.33% after 0.1 µg/ml LPS treatment. When we applied α-syn monomer to the medium, CD200R1 expression decreased significantly, but CX3CR1 expression did not change significantly. The sensitivity of CD200R1 expression under inflammatory conditions may explain why CD200-CD200R1 but not CX3CL1-CX3CR1 decreased in the late stage of PD. Another interesting finding is that the level of cytokine production induced by LPS was nearly ten-fold that induced by α-syn. However, the protein level of CD200R1 decreased to nearly the same level under both treatments. These results indicate that CD200-CD200R1 signaling may contribute to microglial responses other than inflammation, such as phagocytosis or phagoptosis. Further investigation is still needed to determine the mechanism.

## Supplementary Materials

The Supplemenantry data can be found online at: www.aginganddisease.org/EN/10.14336/AD.2019.0615.
