# Supplementary material for: Disease Progression-Dependent Expression of CD200R1 and CX3CR1 in Mouse Models of Parkinson’s Disease
Source: Aging Dis. 2020 Mar 9;11(2):254–68. doi: 10.14336/AD.2019.0615 (PMC7069458; doi:10.14336/AD.2019.0615)
Supplement: Supplementary file 1 — The Supplemenantry data can be found online at: www.aginganddisease.org/EN/10.14336/AD.2019.0615. [file AD-11-2-254-s.pdf]

SUPPLEMENTARY DATA

Disease Progression-Dependent Expression of CD200R1 and CX3CR1 in Mouse Models of Parkinson's Disease

Le Wang[1], Yang Liu[1], Shuxin Yan[1], Tianshu Du[1], Xia Fu[1], Xiaoli Gong[2], Xinyu Zhou[1], Ting Zhang[1,*], Xiaomin Wang[1,2,*]

[1]Department of Neurobiology, Center of Parkinson Disease Beijing Institute for Brain Disorders, Beijing Key Laboratory on Parkinson Disease, Key Laboratory for Neurodegenerative Disease of the Ministry of Education, Beijing Key Laboratory of Neural Regeneration and Repair, Capital Medical University, Beijing, China.
[2]Department of Physiology and Pathophysiology, Capital Medical University, Beijing, China.

© 2019. Wang L et al. Published online at http://www.aginganddisease.org/EN/10.14336/AD.2019.0615

SUPPLEMENTARY DATA

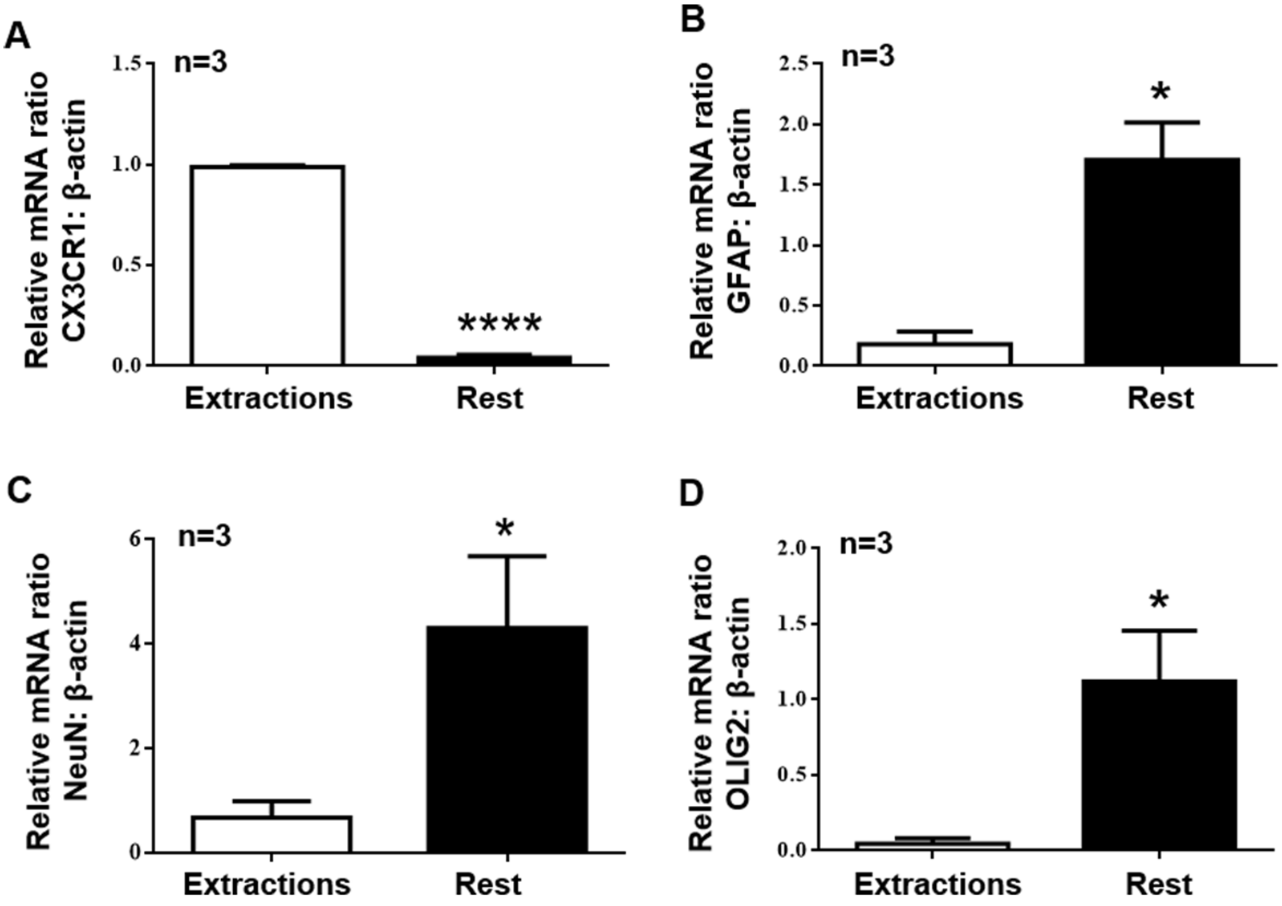

Supplementary Figure 1. **The identification of isolated microglia.** Microglia were isolated from whole brains excluding the cerebellum by immunomagnetic separation. (A, B, C and D) The sorted cells and the rest of the cells were analyzed by RT-qPCR using specific primers. (A) Microglia, *CX3CR1*. (B) Astrocyte, *GFAP*. (C) Neuron, *NeuN*. (D) Oligodendrocytes, *Olig2*. The data are expressed as the mean ± SEM (n = 3 per group). * $p < 0.05$ and *** $p < 0.001$ versus the isolated group, Student's t test.

© 2019. Wang L et al. Published online at http://www.aginganddisease.org/EN/10.14336/AD.2019.0615

SUPPLEMENTARY DATA

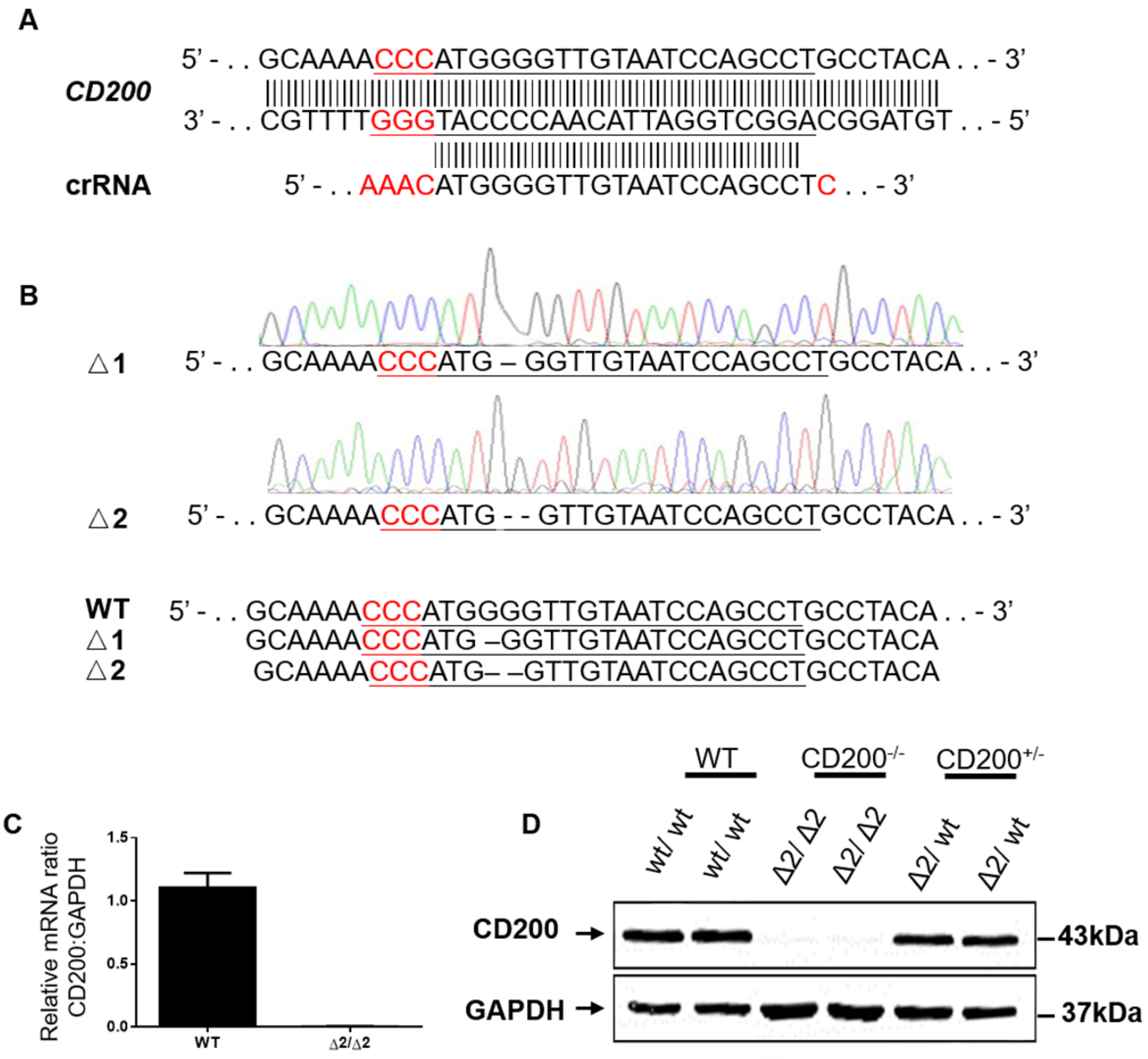

Supplementary Figure 2. The generation and identification of CD200$^{-/-}$ mice. (A) Targeting strategy for the generation of CD200$^{-/-}$ mice by the CRISPR-Cas9 system. (B)The PCR products of the CD200$^{-/-}$ mice were analyzed by direct sequencing, and the wild-type *CD200* gene sequence and the gRNA target sequences (underlined) are shown at the top (WT). The PAM sequence is labeled in red, and the deletions are indicated by dashes. An example chromatogram showing the nucleotide deletions (△1 and △2). (C and D) The identification of CD200 knockout at the transcriptional level (C) by RT-qPCR and at the protein level (D) by Western blotting.

© 2019. Wang L et al. Published online at http://www.aginganddisease.org/EN/10.14336/AD.2019.0615
